# 造血干细胞移植后慢性移植物抗宿主病对死亡影响的统计分析方法辨析

**DOI:** 10.3760/cma.j.cn121090-20250731-00356

**Published:** 2025-10

**Authors:** 俊杰 吴, 俊峰 王

**Affiliations:** 1 荷兰乌得勒支大学，乌得勒支 3584 CS，荷兰 Utrecht University, Utrecht 3584 CS, the Netherlands; 2 荷兰马斯特里赫特大学健康、医学与生命科学学院，马斯特里赫特 6229 HA，荷兰 Department of Epidemiology, CAPHRI Care and Public Health Research Institute, Maastricht University, Maastricht 6229 HA, the Netherlands

**Keywords:** 慢性移植物抗宿主病, 时间依赖性事件, 生存分析, Chronic Graft-versus-host Disease, Time-dependent Events, Survival Analysis

## Abstract

**目的:**

以造血干细胞移植后慢性移植物抗宿主病（chronic graft-versus-host disease，cGVHD）对总生存率的影响为示例，介绍和辨析生存分析中处理时间依赖性事件的5种常用方法。

**方法:**

基于国际血液和骨髓移植研究中心（CIBMTR）GV18-03研究，纳入2008至2017年间资料完整、年龄≥40岁、接受HLA 8/8同胞全相合移植的急性髓系白血病或骨髓增生异常综合征患者4 361例。分别将cGHVD作为基线固定变量（方法1）、作为时间依赖性协变量（方法2）、通过界标时间划分（方法3）、考虑为多状态模型（方法4）、应用参数g-formula法（方法5）进行分析。展示每种方法的核心思路和对应的分析结果，阐述方法的原理、优劣与适用情境。

**结果:**

不同方法在偏倚控制、建模灵活性及临床解释性方面存在显著差异。方法1易导致未亡时间偏倚；方法2能在一定程度上校正偏倚，但面临非随机删失及前期cGVHD组样本量不足，导致估计不稳定的问题；方法3简明直观，但依赖人为提前设定的界标时点，且无法处理界标时间后才发生的cGVHD；方法4适用于临床状态动态转移过程的模型构建，能全面描述时间依赖性事件对生存预后的影响；方法5可同时处理时间依赖性事件与时间依赖性混杂，适合估计特定“暴露策略”（特定时间依赖性事件发生模式）的群体效应。

**结论:**

研究者应结合具体研究目标，选用适当方法，确保对时间依赖性事件对结局的影响的估计具备统计学有效性与临床解释的合理性。

## Abstract (en)

**Objective:**

Using the impact of chronic graft-versus-host disease（cGVHD）on overall survival after hematopoietic stem cell transplantation as an example, this study aims to introduces and critically appraises five statistical approaches for handling time-dependent events in survival analysis.

**Methods:**

This study was based on data from the Center for International Blood and Marrow Transplant Research（CIBMTR）GV18-03 study. A total of 4361 patients with acute myeloid leukemia or myelodysplastic syndrome were included, who were aged ≥40 years and had received an HLA 8/8 matched sibling donor transplant between 2008 and 2017. Five analytical approaches were used, treating cGVHD as: ① a baseline fixed covariate, ② a time-dependent covariate, ③ a time-dependent event via landmarking analysis, ④ a transitional event in a multi-state model, and ⑤ an exposure in the parametric g-formula. For each approach, we presented its core concept and results, and elaborated on its rationale, strengths, limitations, and applicable scenarios.

**Results:**

The five approaches showed significant differences in bias control, modeling flexibility, and clinical interpretability. Method 1 was prone to immortal time bias. Method 2 partially corrected this bias but was vulnerable to informative censoring and estimation instability due to insufficient sample sizes in the early-onset cGVHD group. Method 3 was straightforward but dependent on a predefined landmark time point and unable to account for cGVHD occurring after this point. Method 4 allowed for a comprehensive description of the impact of time-dependent events on survival prognosis by modeling dynamic clinical transitions. Method 5 could simultaneously handle both time-dependent events and confounders, making it suitable for estimating population-level effects of specific exposure strategies.

**Conclusion:**

Researchers should select the appropriate method based on their specific study objectives to ensure that the estimation of the impact of time-dependent events on outcomes is both statistically valid and clinically meaningful.

临床研究中常会发生某些随时间发生的中间事件，例如特定疾病状态的发生或对治疗干预方案的调整。这类事件并非从研究起始时就确定，而是在随访过程中动态变化，因此被称为时间依赖性事件。此类事件不仅发生与否很重要，其发生的时间早晚也同样可能影响结局。对时间依赖性事件的统计处理方式直接影响研究推断与结论的有效性，将其视为基线时就存在的固定变量进行分析就会引入未亡时间偏倚（immortal time bias）[1]–[2]，从而扭曲结果的估计。Agarwal等[3]利用真实数据和模拟分析发现，不合理地处理时间依赖性治疗（治疗状态随时间改变）会导致结果有显著偏倚。

慢性移植物抗宿主病（chronic graft-versus-host disease，cGVHD）是一种异基因造血干细胞移植后常见的中间事件，对患者预后有显著影响[4]–[5]。患者后续会不会发生cGVHD并不是在移植当天就能知道，其发生时间及持续过程通常在移植后随时间记录。因此，在分析中必须把它当作一个时间依赖性事件处理。如果忽略这一特性，往往会导致研究结论出现偏差。

鉴于此，为避免因方法选择不当而得出误导性结论，研究者须掌握在生存分析数据中处理这类随时间变化事件的恰当方法。选择方法时应当结合各自研究目的，从而保证研究分析结果更符合客观规律。为了帮助研究者系统地理解不同方法的原理、优劣与适用情境，本文梳理并介绍了生存分析中处理时间依赖性事件的5种常见方法：①作为基线固定变量（方法1）；②作为时间依赖性协变量（方法2）；③界标分析法（landmarking analysis）（方法3）；④多状态模型（multi-state model）（方法4）；⑤参数g-formula法（parametric g-formula）（方法5）。其中，方法1在统计学上是错误的做法，作为反例列出。方法2和方法3虽然被广泛应用，但各自存在明显的缺陷。方法4和方法5更能反映时间依赖性事件随时间演变的动态特征，是生存分析中较为前沿的方法。理解这些不同的方法并能够实践应用，有助于临床研究人员更准确地估计这类时间依赖性事件对生存预后的影响，进而辅助临床决策。

本文以真实的造血干细胞移植队列数据作为案例，围绕cGVHD这一时间依赖性事件，循序渐进地介绍并应用上述5种分析方法，展示其在估计总生存（OS）方面的差异，并提供完整的R语言代码，供读者复现和后续相关研究参考。

## 数据来源

示例数据集基于国际血液和骨髓移植研究中心（the Center for International Blood and Marrow Transplant Research, CIBMTR）注册研究（编号：GV18-03）。GV18-03数据集纳入了在2008至2017年间，年龄≥40岁，为治疗急性髓系白血病（AML）或骨髓增生异常综合征（MDS），首次接受HLA 8/8同胞全合移植的4 365例患者。该研究旨在探索在年龄≥60岁的患者中，cGVHD的发生是否增加患者非复发死亡风险[6]。

本研究主要研究的变量包括：随访时间、是否死亡（终点事件，二分类变量）、cGVHD发生时间、cGVHD状态（二分类变量）、基线特征（年龄、性别、供受者性别匹配）。随访时间定义为移植至死亡或末次随访的时间，cGVHD的发生被视为移植后的中间事件，其特点是随时间动态变化，因此在分析中应作为时间依赖性事件处理。

本研究将所有患者的随访时间统一截断于120个月。排除研究变量存在缺失的患者后，共纳入4 361例患者进入后续分析。纳入的患者中位OS时间为24.74（95％*CI*: 22.73～26.84）个月。随访期间共有2 335例患者在死亡或失访前发生了cGVHD。在考虑死亡为竞争风险事件的情况下，cGVHD在12个月和24个月的累计发病率分别为（45.79±0.76）％和（52.00±0.76）％，图1展示了其在0～24个月内的cGVHD累计发生率曲线。表1展示了患者的基线特征。需要指出的是，为保持与原始研究的一致性，本研究纳入了AML与MDS两类患者，在数据上存在一定异质性。在实际研究中应满足数据同质性的统计前提，建议对病种作相应的区分然后进行分析。特别说明，本文中的案例分析与展示仅作为方法学上的实践示例，不应从展示的结果中推导任何临床相关结论。

**图1:**
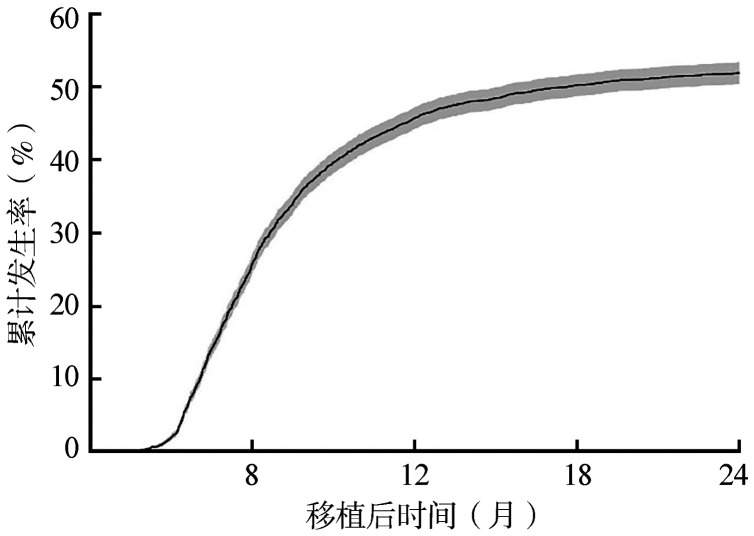
GV18-03研究中患者异基因造血干细胞移植后0～24个月慢性移植物抗宿主病的累计发生率曲线（考虑死亡为竞争风险事件）

**表1:** GV18-03研究中接受异基因造血干细胞移植治疗急性髓系白血病与骨髓增生异常综合征4 361例患者基本信息

变量	例数（％）
年龄	
40～49岁	678（15.5）
50～59岁	1 436（32.9）
60～69岁	1 864（42.7）
≥70岁	383（8.8）
性别	
男性	2 572（59.0）
女性	1 789（41.0）
供受者性别匹配	
供受者性别错配	1 930（44.3）
供受者性别一致	2 431（55.7）

## 案例分析

一、将cGVHD状态作为基线固定分组变量（方法1）

如果想要研究cGVHD对于死亡的影响，首先想到的方法，便是将患者按cGVHD状态分组，即将所有发生过cGVHD的患者归为一组，未发生cGVHD的患者归另一组。然后，将cGVHD状态当作一个基线信息，分别绘制Kaplan-Meier生存曲线比较两组生存率，并使用Cox比例风险模型进行回归分析。已有研究采用此方法，按cGVHD状态分组后比较无病生存率，结果显示“发生cGVHD组”患者的无病生存率显著高于“未发生cGVHD组”[7]。

图2是运用方法1得到的Kaplan-Meier生存曲线，显示“未发生cGVHD组”的生存概率显著低于“发生cGVHD组”。Cox风险比例模型结果呈现在表2，显示“发生cGVHD组”的死亡风险显著低于“未发生cGVHD组”。

**图2:**
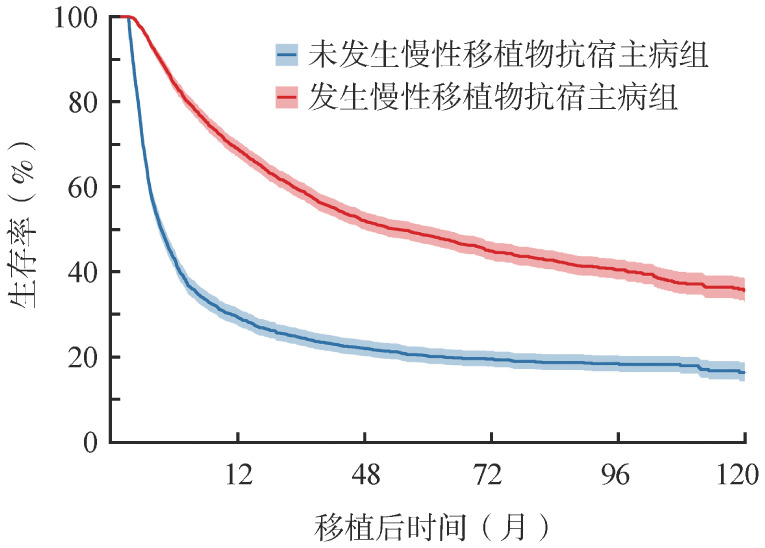
以是否发生慢性移植物抗宿主病为基线固定分组变量的异基因造血干细胞移植患者0～120个月总生存Kaplan-Meier曲线

**表2:** 利用5种方法分析移植后慢性移植物抗宿主病对总生存影响的风险比估计值及其置信区间

方法	*HR* ^c^	95％ *CI*
基线固定变量（方法1）	0.38	0.35～0.40
时间依赖性协变量（方法2）	0.79	0.73～0.86
界标分析法（方法3）		
6个月为界标	0.94	0.85～1.03
12个月为界标	0.93	0.83～1.03
24个月为界标	1.12	0.95～1.32
多状态模型（方法4）^a^	NA	NA
参数g-formula法（方法5）^b^	0.87	0.82～0.94

**注** ^a^在多状态模型中，cGVHD被视为一个中间状态，其对预后的影响通过转移概率来体现，不直接提供*HR*估计；^b^参数g-formula法得到的是边际*HR*，其95％*CI*基于Bootstrap方法计算；c：本表中方法所得的*HR*以“未发生cGVHD组”为参考组；NA：不适用

尽管这种做法直观且易操作，但在统计学上存在明显错误：将随时间变化的cGVHD状态即时间依赖性变量（time-dependent variable）作为基线固定变量（time-independent variable）[8]。这样的分组方式在生存分析中是错误的，因为患者必须要在移植后存活到足够长的时间才有可能“发生cGVHD”而进入该组，而那些较早死亡的高风险患者则会进入“未发生cGVHD组”。

对于最终发生了cGVHD的患者，其在cGVHD发生前不可能死亡，这段尚未发生cGVHD的时间区间就是所谓的未亡时间（immortal time）。处于未亡时间中的患者实际仍处于未发生cGVHD的状态，但在该分析方法中却在基线时刻提前被错误地分至“发生cGVHD组”并计算入该组生存时间，导致系统性地高估了该组的生存概率。这样分析处理方式引入的偏倚在方法学中被称为未亡时间偏倚[1]–[2]。

二、将cGVHD状态作为时间依赖性事件（方法2）

将cGVHD状态作为时间依赖性协变量进行分析，相比作为基线变量更为合理。这种处理方式既可用于生存曲线分析，也可在Cox比例风险模型中进行回归分析。例如，一项针对急性淋巴细胞白血病（ALL）患者的研究中，便采用了方法2分析GVHD对OS的影响[9]。

本研究中，我们选择“早期起始时间点”为3个月，图3为该方法绘制的Simon-Makuch图。在绘图过程中，1例患者在发生cGVHD之前是属于“未发生cGVHD组”曲线；当cGVHD发生时，该患者会马上归于“发生cGVHD组”并计入相应曲线。相较于方法1，“未发生cGVHD组”与“发生cGVHD组”的生存曲线明显更加接近。时间依赖Cox风险比例模型结果（表2）显示，“发生cGVHD组”的死亡风险显著低于“未发生cGVHD组”，但两组差异减小（相比于方法1的结果，方法2得到的*HR*更接近1）。这些结果表明，方法2在一定程度上减弱了“未亡时间偏倚”对组间比较结果的影响。

这里不采用Kaplan-Meier方法，是因为其不适用于包含时间依赖性协变量的生存曲线绘制。而Simon-Makuch方法恰好适用于这种情况，其核心思想是：当协变量状态随时间发生变化时，将原始的单行个体数据按协变量状态拆分为多行，以反映个体在不同时段内的状态变化。本质上，这是一个动态调整风险集（riskset）的方法，在每一个时间依赖性事件发生时点根据当前变量状态进行风险集动态更新[10]–[11]。

**图3:**
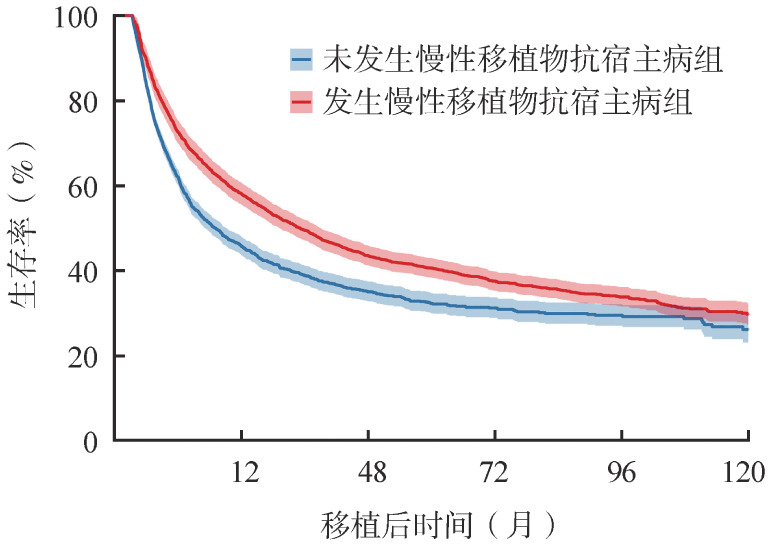
以慢性移植物抗宿主病状态为时间依赖性变量的异基因造血干细胞移植患者3～120个月总生存Simon-Makuch曲线

需要注意的是，Simon-Makuch方法建议人为设定“早期起始时间点”。这是为解决随访初期“发生cGVHD组”为空，风险率无法在最早的cGVHD发生时间之前计算的问题，同时可以提高生存估计的稳定性。在此时间点进行分组，可确保各组在起始时具有足够数量的风险个体。但该起点之前发生的终点事件将被忽略，因此延迟起始时间点的选择需结合终点事件的实际发生分布情况来权衡[10]。

虽然与方法1相比，方法2在一定程度上解决了“未亡时间偏倚”，但是方法2也存在局限性。一方面，由于患者一旦发生cGVHD就会被划归“发生cGVHD组”，在原“未cGVHD发生组”将其视为删失，违反了随机删失的基本假设（因为cGVHD的发生显然不是随机的）。另一方面，在研究初期，“发生cGVHD组”的风险人数可能偏少，若在某一时间点该组内所有患者均发生终点事件，该组生存率将降至0，并在后续时间内保持不变，也就是说即使后来仍有患者加入该组，也无法改变其生存曲线[10]。

三、界标分析法（方法3）

利用界标分析法，可以进行Kaplan-Meier曲线绘制和Cox比例风险模型回归分析。已有研究采用此方法，探索了在AML、ALL等疾病患者中，移植后1年存活者cGVHD的发生对晚期复发的影响[12]。

本研究中设置的界标时间分别为移植后6、12和24个月，根据在该时间点之前是否发生cGVHD，将患者划分为两组：在6、12和24个月内发生cGVHD且仍存活者归入“发生cGVHD组”，在6、12和24个月内未发生cGVHD且仍存活者归入“未发生cGVHD组”。图4显示，“发生cGVHD组”与“未发生cGVHD组”的生存概率整体相近。Cox风险比例模型结果呈现在表2，显示两组间死亡风险差异不显著。

**图4:**
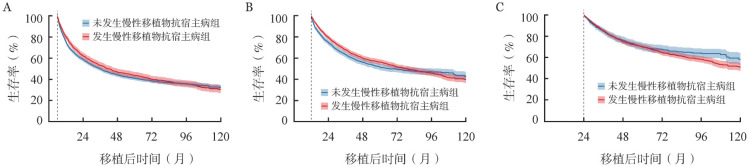
设置界标时间分析是否发生慢性移植物抗宿主病的异基因造血干细胞移植患者界标时间至120个月总生存Kaplan-Meier曲线 **A** 以移植后6个月为界标；**B** 以移植后12个月为界标；**C** 以移植后24个月为界标

界标分析法是通过设定一个固定时间点将时间依赖性协变量转化为固定协变量。根据该固定时间点之前是否发生cGVHD，将患者划分为两组：在该时间点前发生cGVHD且仍存活者归入“发生cGVHD组”，在该时间点前未发生cGVHD且仍存活者归入“未发生cGVHD组”。

该方法操作简便，且在一定程度上缓解“未亡时间偏倚”，研究者可直接应用经典生存分析方法。与方法2中将cGVHD作为时间依赖性协变量不同，界标分析法满足随机删失的前提假设。

不过，界标分析法还是存在局限性。首先，是否发生cGVHD的分组基于选定的界标时间，在界标时间前已发生终点结局（如死亡）的患者将被排除在分析之外，并且只有“存活足够长”至界标时间点的患者才能用于分析，这样的处理可能引入偏倚。其次，该方法没有考虑界标时间之后发生的cGVHD对结局的影响。

除上述局限之外，如何设置时间标记点也是一项难题。因为设置不同的点，会对分析的人群以及最后的结果有很大的影响。若设定界标时间点过早，仅有少部分患者发生了cGVHD，这会导致组间样本数不均衡。而设置得过晚，则会显著减少可纳入分析的样本量。这两种情形均可能导致检验效能降低（loss of power）。因此，研究者需要结合研究目的与事件（cGVHD和终点事件）发生分布，选择一个合适的界标时间点[13]–[14]。

四、多状态模型（方法4）

多状态模型可通过定义有限的多个状态与其转移路径，对患者不同临床状态的随机转移进行建模，从而动态描述疾病演进过程，适用于含有时间依赖性事件（如cGVHD）的生存分析[15]–[17]。既能保留全部的随访信息，又能动态反映cGVHD的发生对死亡的影响。

本示例中，患者初始处于移植后无病状态（状态1），可转移至发生cGVHD状态（状态2），或直接转移至死亡状态（状态3）；若进入发生cGVHD状态，亦可能进一步发展为死亡状态。在多数移植相关研究中，中间事件的处理通常不考虑状态逆转，即不考虑发生cGVHD的患者（状态2）回到无病状态（状态1），当然患者也不可能死而复生（即状态3为吸收状态）（图5）。

**图5:**
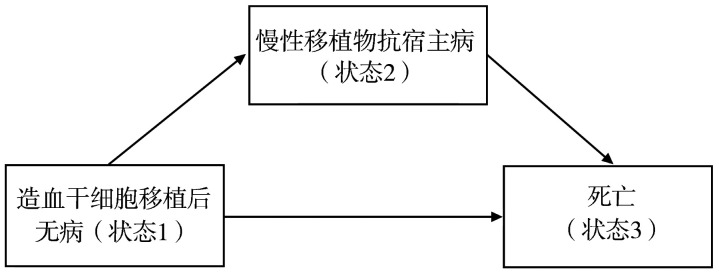
异基因造血干细胞移植后发生慢性移植物抗宿主病的多状态模型临床状态转移示意图

值得一提的是，看到图5时，部分熟悉生存分析研究者可能会联想到竞争风险模型。实际上，竞争风险模型是多状态模型的一种特例，它不考虑中间状态到终点状态的转移，而是将示例中两个状态视为两个互相竞争的终点事件。其优势在于能够同时处理多个终点，但其分析的重点仅限于最先发生的事件，因此只能估计如“在死亡前发生cGVHD”的概率。然而，我们的研究需要回答：cGVHD作为中间事件，它的发生会如何影响死亡？竞争风险模型无法直接解决这一问题。

多状态模型建模可以采用R语言中的“mstate”（版本0.3.3）。图6展示患者自移植起，状态随时间变化的概率分布。图7中左列为患者分别自移植后6、12与24个月，从状态1开始的状态转移概率曲线；右列为相同时间点已进入状态2（发生cGVHD）后的状态变化。在移植后6个月时，尚未发生cGVHD的患者其后续5年内（即至移植后5.5年）的死亡概率为61％，高于同期已发生cGVHD患者的死亡概率（56％）。在移植后12个月时，尚未发生cGVHD患者的5年内（即至移植后6年）死亡概率为50％，亦略高于已发生cGVHD患者的死亡概率（48％），但二者差异有所缩小。至移植后24个月，情况发生逆转，尚未发生cGVHD患者的后续5年（即至移植后7年）死亡概率降至35％，低于已发生cGVHD患者的死亡概率（39％）。

**图6:**
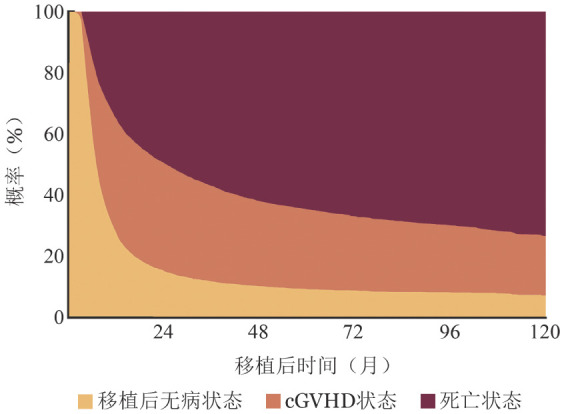
基于多状态模型的异基因造血干细胞移植患者移植后0～120个月状态概率堆积图

**图7:**
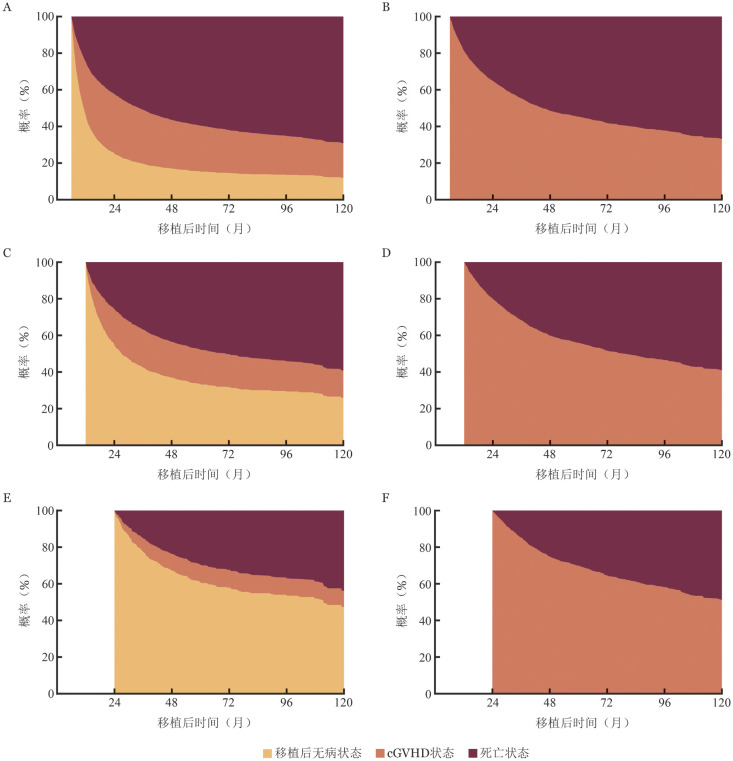
基于多状态模型的异基因造血干细胞移植患者移植后以6、12和24个月为起点的状态概率堆积图 左列图展示了以移植后分别以6个月（A）、12个月（C）和24个月（E）为起点时，患者从移植后无病状态开始，随时间变化处于各个状态的概率；右列图则展示了以移植后6个月（B）、12个月（D）和24个月（F）为起点时，患者以进入慢性移植物抗宿主病状态为起始点，随时间变化处于各状态的概率

多状态模型的建模流程一般包括以下几个步骤（1）设定转移矩阵：定义允许的状态转移路径；（2）拟合模型：对每一条状态转移路径分别构建Cox比例风险模型（无协变量），允许各路径拥有独立的基线风险；（3）估计转移强度；（4）计算转移概率：利用已估计的转移强度，计算不同时间点从当前状态转移至其他状态的概率；（5）可视化：绘制状态堆积图，动态展示患者状态随时间变化的概率分布。

在实际运用中，马尔科夫（Markov）模型因其简便性被广泛采用[17]。所谓Markov性，是指风险仅依赖于当前时间和状态，与过去时间和转移状态无关的性质[18]。如果该假设不成立（例如风险受当前状态持续时间影响），则需采用更复杂的半Markov模型或非Markov模型。在此类模型中，可引入“持续时间”作为协变量，或通过设置多时间尺度进行调整，相关方法可参考文献[17],[19]。

多状态模型不仅能明确展示随访开始后任一时点患者在不同状态间转移的概率，还能提供在某一特定时间点后，处于不同临床状态的患者的后续状态转移情况，相当于解决了界标分析法（方法3）不考虑界标时间点后cGVHD发生的缺陷，更全面反映疾病动态演进过程。

五、参数g-formula法（方法5）

参数g-formula法是一种用于处理观察性研究中时间依赖性暴露与时间依赖性混杂因素的方法，可用于估计任意暴露策略下的潜在结局期望，并通过比较不同策略得到暴露的效应[20]。对于时间依赖性事件（如cGVHD）的研究，其原理同样适用。时间依赖性事件的发生模式可视作一种特殊的“暴露策略”，参数g-formula法可以模拟该事件“永远不发生”、“一开始就发生”或“当某协变量低于一定值时发生”等情境下的潜在结局，来估计时间依赖性事件对结局的影响。

示例中，我们采用R软件中的“gfoRmula”包[21]（版本1.1.1）构建参数g-formula模型。为了简化模型，仅将cGVHD状态设为唯一随时间变化的变量，且一旦发生即保持不变。在建模过程中，首先拟合cGVHD状态的变化模型；然后在结局模型中纳入患者的基线协变量、时间变量、cGVHD当前状态变量和上一时点cGVHD状态变量。结果展示在表2。需要注意的是，与传统方法所输出的条件*HR*不同，参数g-formula法估计的是边际*RR*和边际*HR*[22]–[23]，并且由于*HR*具有非坍缩性（non-collapsibility）[24]，即便不存在混杂因素，边际*HR*与条件*HR*之间也可能存在差异。但为保证组间比较的效应指标的一致性，本文仍在表2中采用*HR*作为效应指标。

传统化方法无法处理随时间变化的协变量，而g-formula法是传统方法在存在时间依赖性协变量情境下的拓展；相较之下，g-formula法在大量协变量存在的情况下使用受限制，而参数g-formula法则是g-formula法在这个情境下的拓展[25]。具体操作中，在满足“无未测量混杂因素、无测量误差，且无模型错误设定”的假设下，对每个时间点，参数g-formula法可以利用预设的cGVHD参数模型（包含基础协变量和上一个时间点cGVHD状态信息）估计当前时点发生cGVHD的条件概率。随后，利用预设的结局参数模型可以估计当前时间点的死亡风险。当然这只是方法的第一步，后续还需要利用蒙特卡洛模拟，得到完整随访路径，从而可以生成预设的不同cGVHD发生模式（如自然病程、一开始就患cGVHD和永不患cGVHD）下大量个体的结局，最终得到不同cGVHD发生模式下的死亡风险[20],[22]。

参数化g-formula法具有高度灵活性，既可正确处理时间依赖性暴露（事件），也可避免由既是混杂因素又是中介变量的时间依赖性变量所引入的偏倚[23]。不仅如此，该方法还可以用于估计复杂时间依赖性事件的发生模式下（如动态策略，随机策略等）潜在结局期望[21]。但是该方法需要构建多个模型参数，所以特别依赖于模型正确设定[23]。

六、方法总结

在移植后cGVHD对预后影响的研究中，将cGVHD简单地视作为基线固定分组变量（方法1），在统计方法上属于本质性错误，因其忽略了cGVHD发生的时间信息，等同于假设研究起始阶段就已知其是否发生，错误处理事件发生的时间顺序，容易引入严重的未亡时间偏倚。相比之下，方法2与方法3在一定程度上可以校正此类偏倚。将cGVHD作为时间依赖性协变量进行分析（方法2），考虑了cGVHD发生的时序信息，是校正此类偏倚的常用方法。该方法可直接估计*HR*，并可配合Simon-Makuch绘图展示随时间变化的生存曲线，相较于方法1可以提供更合理的估计。界标分析法（方法3）更直观易懂，也同样遵循了事件发生的时间顺序，可回答“若患者存活至移植后某一时间点，该时点是否患有cGVHD对生存预后有何影响”。但分析结果高度依赖界标时点的设定，且无法纳入界标时间之后才发生的cGVHD事件，可能低估其真实影响。多状态模型（方法4）具有明显优势，可呈现患者自随访开始后不同时间点各临床状态之间的转移概率，还避免了界标分析法（方法3）中无法分析界标时间点后发生的cGVHD的局限性。另外，多状态模型（方法4）还允许临床研究者设定其认为具有代表性的、有研究价值的特征人群，利用该方法对特征人群的状态转移和生存预后进行预测[21]。参数g-formula法（方法5）适用于存在时变“暴露”（本研究情境下的时间依赖性事件）和时变混杂的情况，可以结合个体历史信息估计某些特定时间依赖性事件发生模式在群体水平对结局的影响，如设定模拟估计“所有人从不发生cGVHD”、“所有人从一开始就发生cGVHD”或“所有人从移植后半年起发生cGVHD”等发生模式下的10年生存概率，回答“如果控制cGVHD的发生时间，会如何影响生存预后”的问题。相应方法的对比和总结，呈现在表3。为方便读者复现本文的分析，案例分析部分中使用的R语言代码已提供在https://github.com/JunjieWu1999/Time-Dependent-Events-in-Survival-Analysis_cGVHD。

**表3:** 生存分析中处理时间依赖性事件的5种常用方法信息总结表［以移植后发生慢性移植物抗宿主病（cGVHD）为例］

方法	图示	统计模型	所用R包	优点	缺点
方法1：cGVHD为基线固定变量	Kaplan-Meier曲线	Cox比例风险模型	“survival”	无	统计学典型错误，未亡时间偏倚
方法2：cGVHD为时间依赖性事件	Simon-Makuch曲线	Cox比例风险模型	“survival”	一定程度上校正“未亡时间偏倚”	①非随机删失；②研究初期发生cGVHD组人数少，可能发生生存概率为0，而后续不变的情况
方法3：界标时间点处分组	界标Kaplan-Meier曲线	Cox比例风险模型	“survival”	①一定程度上缓解“未亡时间偏倚”； ②操作简便直接应用经典生存分析方法	①界标时间前已发生终点事件的患者将被排除在分析之外； ②没有考虑界标时间之后发生的cGVHD对结局的影响； ③界标时间的选择影响结果
方法4：多状态模型	状态概率堆积图	多状态模型	“mstate”	展示任一时点后患者在不同状态间转移的概率，全面反映疾病动态过程	cGVHD已变成中间状态，不直接提供*HR*估计
方法5：参数g-formula法	无	参数g-formula法	“gfoRmula”	①高度灵活； ②可处理时间依赖性暴露（事件）和混杂； ③可用于复杂cGVHD发生模式	准确性依赖于正确的模型设定

除上述所列方法外，尚有边际结构模型联合逆概率加权（inverse probability weighting of marginal structural models）和结构嵌套模型联合g-estimation（g-estimation of structural nested models）等其他g-method方法可用，因超出本文范围故未作展开。此外，参数g-formula法存在g-null悖论（g-null paradox），即当零假设为真实效应为零时，该方法会错误地拒绝零假设。但与随机变异相比g-null悖论引入的偏倚较小，并不会阻碍实际应用[26]。

## 讨论

本文采用以点带面的形式，以真实的临床研究数据为例，介绍了处理cGVHD这类时间依赖性变量的5种常用方法。值得指出的是，在造血干细胞移植相关研究中，除cGVHD外，还有很多其他变量也具有时间依赖性特征，同样可以借鉴本文中介绍的统计方法进行处理。例如，在一项针对慢性粒-单核细胞白血病（CMML）患者接受异基因造血干细胞移植是否可以提高患者OS率的研究中，在确诊CMML至死亡终点事件之间，造血干细胞移植作为一种时间依赖性事件，使用多状态模型进行分析[27]；在另外一项关注复发时间对套细胞淋巴瘤（MCL）患者自体造血干细胞移植后OS的影响的研究中，在移植后随访期间的复发也属于随时间动态变化的事件，使用了界标分析的方法进行分析[28]。上述情景与cGVHD类似，即事件并非在基线时固定，而是在随访过程中发生或变化，提示研究者在设计与分析中应特别关注这些事件发生的时点与动态变化过程。

综上所述，笔者建议研究者应该根据具体的临床研究问题，选用合适的分析方法。如果需要研究疾病复杂的状态转移过程，就可以考虑采用多状态模型。当研究目的是需要估计特定时间依赖性事件发生模式的群体平均效应时，需要基于有向无环图[29]明确时间依赖性事件、协变量和结局之间的关系，并且谨慎地构建关于时间依赖性变量和结局的一系列参数模型，在此基础上运用参数g-formula法。对于上述所有方法，务必结合研究目标合理选择，这样以来才能确保在统计学上时间依赖性事件效应的有效估计和临床上相关结果解释的合理。作为实践示范，本研究展示了存在时间依赖性事件的情景下，如何采用合理的方法正确分析并估计该类事件对OS率的影响，为当前国内研究人员解决相关问题提供了有益参考。
